# Supplementary material for: Feedback-controlled active brownian colloids with space-dependent rotational dynamics
Source: Nat Commun. 2020 Aug 24;11:4223. doi: 10.1038/s41467-020-17864-4 (PMC7445303; doi:10.1038/s41467-020-17864-4)
Supplement: Supplementary file 1 — Supplementary Information [file 41467_2020_17864_MOESM1_ESM.pdf]

Feedback-Controlled Active Brownian Colloids with Space-Dependent Rotational Dynamics — Supplementary Information

Miguel Angel Fernandez-Rodriguez,[1,*] Fabio Grillo,[1,*] Laura Alvarez,[1]
Marco Rathlef,[1] Ivo Buttinoni,[1,2] Giovanni Volpe,[3] and Lucio Isa[1]

[1] *Laboratory for Soft Materials and Interfaces, Department of Materials, ETH Zurich, 8093 Zurich, Switzerland*

[2] *Institut für Experimentelle Kolloidphysik, Heinrich-Heine University, 40225 Düsseldorf, Germany*

[3] *Department of Physics, University of Gothenburg, 41296 Gothenburg, Sweden*

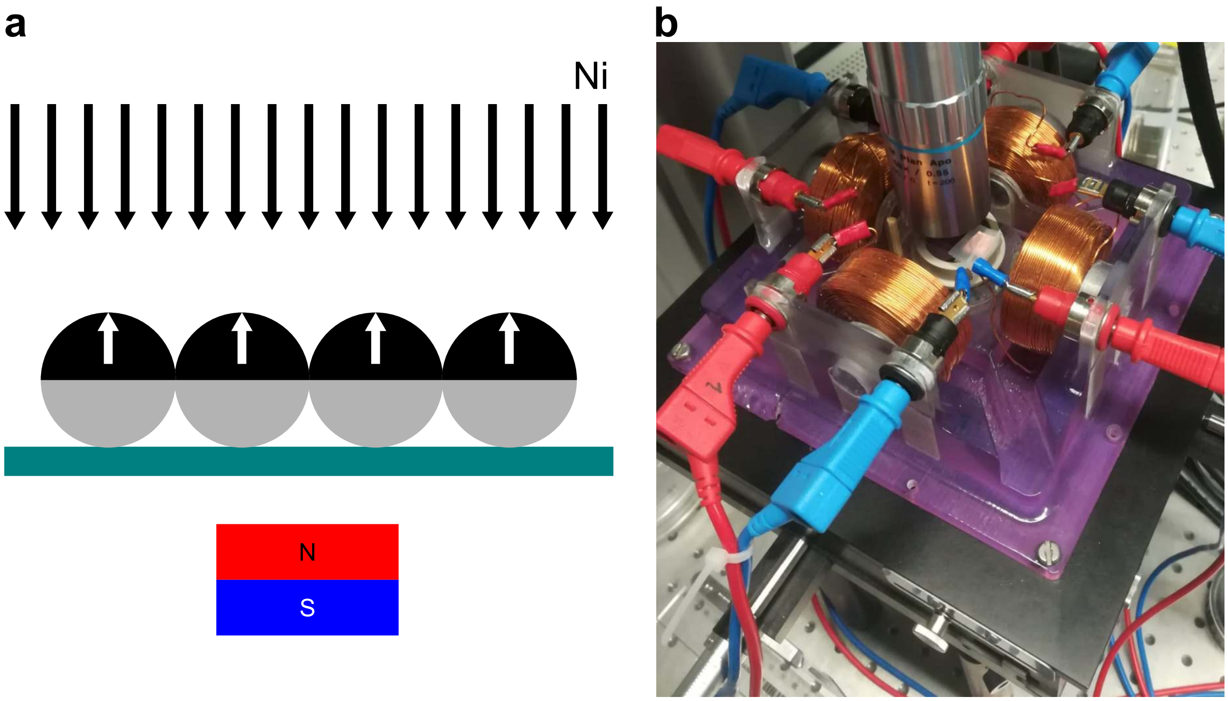

Supplementary Figure 1. Janus particle fabrication procedure and experimental setup. **a** Schematics of the process used to deposit 120 nm of nickel on a self-assembled monolayer of silica particles and obtain Janus particles with magnetic moments that are aligned with the cap's orientation. **b** Photograph of the experimental setup showing the 4 coils and the conductive cell used to apply the AC field.

Size	D_R^{th} (s^{-1})	D_T^{th} (μ m^2 s^{-1})
4 μm	0.014 ± 0.001	0.055 ± 0.002
2 μm	0.21 ± 0.02	0.15 ± 0.002

Supplementary Table 1. Thermal rotational and translational diffusion coefficients of 2 μm and 4 μm Janus particles measured close to the substrate.

* These authors contributed equally to this work

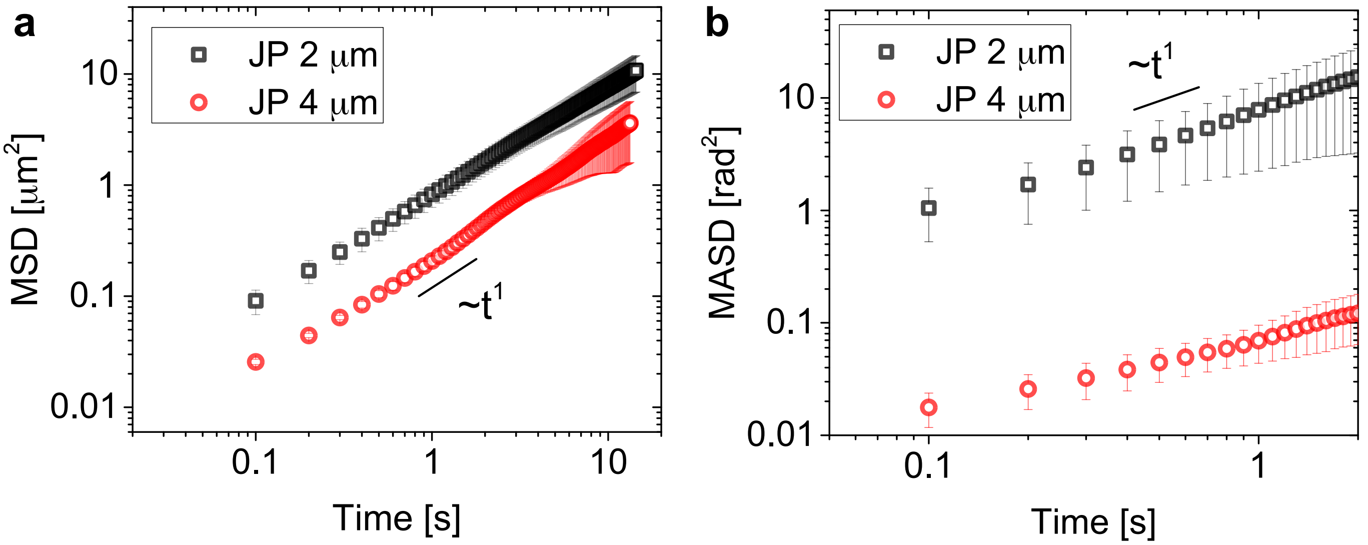

Supplementary Figure 2. Diffusion without AC and magnetic fields applied. **a** Mean squared displacement over time for the 4 μm (red circles) and 2 μm (black squares) JPs. **b** Mean angular squared displacement over time for the 4 μm (red circles) and 2 μm (black squares) JPs.

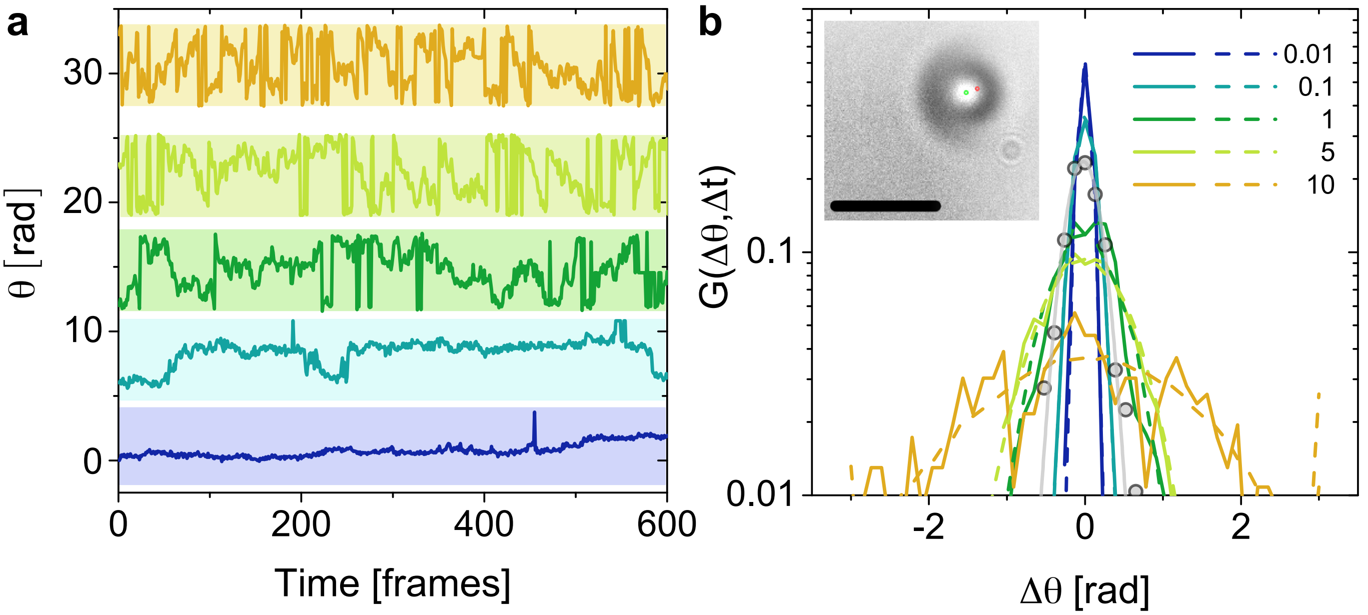

Supplementary Figure 3. Controlling the rotational dynamics of 2 μm particles. **a**Imposed orientation angle vs. time for 2 μm particles with imposed values of D_R=0.01, 0.1, 1, 5, 10 s^{-1} (from the bottom to the top). The colored bands delimit a 2π range. Data are shifted along y for clarity. **b** Probability distributions of measured angular displacements $G(\Delta\theta, \Delta t)$ for different D_R (dashed lines: imposed $\Delta\theta$; solid lines: measured $\Delta\theta$) with the same colors as in **a**. The grey points show $G(\Delta\theta, \Delta t)$ for the thermal D_R^{th} at room temperature, with the field off (grey line: Gaussian fit, see Supplementary Table 1).

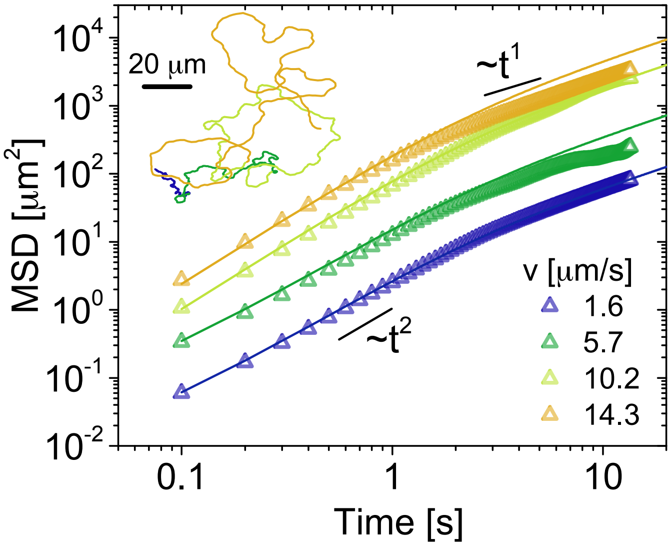

Supplementary Figure 4. Trajectory and mean square displacement of a $4\,\mu m$ JP with a constant D_{R} $1\mathrm{s}^{-1}$ for a step-wise increase of propulsion velocities: 1.6, 8.5, 10.2, and 14.3 μm s^{-1}. As can be seen from the MSDs, in this case the behaviors of the particle are just rescaled versions of each other, as opposed to the qualitative changes we observe as a result of changing $\mathrm{D_R}$ while keeping v constant (Figure 1e).

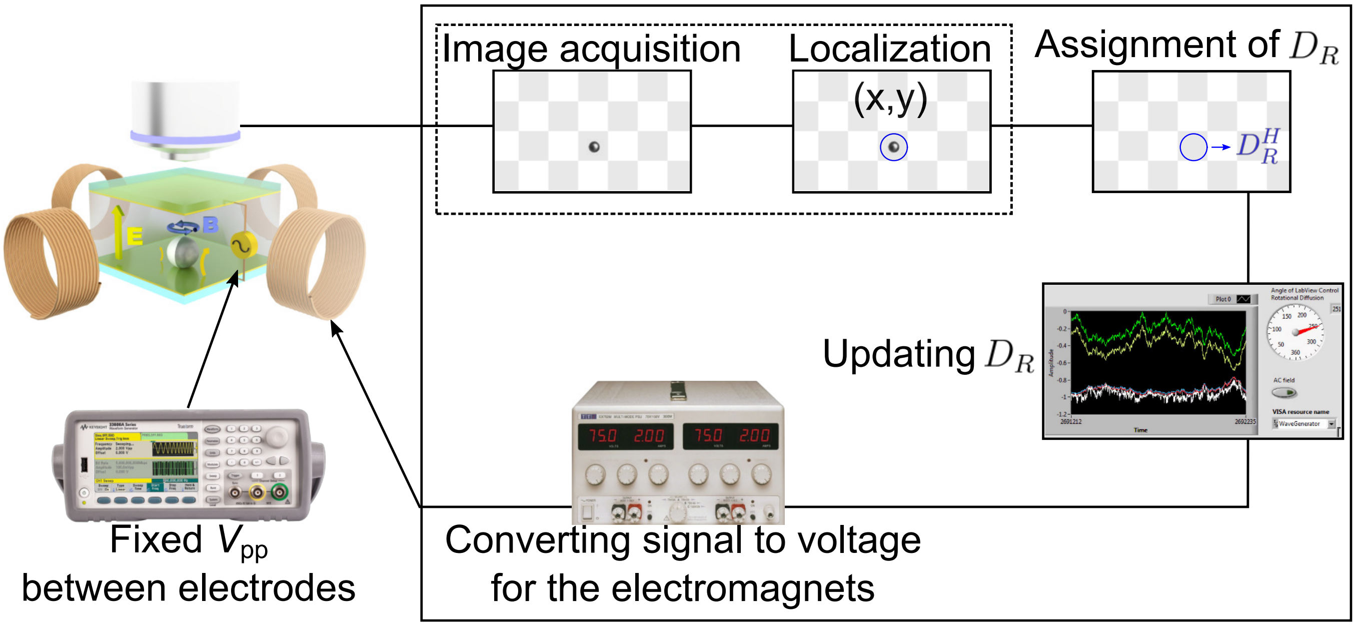

Supplementary Figure 5. Scheme of the experimental discrete-time feedback loop used to update D_{R} based on the position of the Janus particle. The tasks in the control loop are represented within the black rectangle. The time-limiting steps are the image acquisition and particle localization, enclosed in the dashed rectangle, which take 170ms and determine the minimum τ. The signals for the coils are generated at 1000 Hz. Different values of τ are generated by updating the value of D_{R} every n frames, such that $\tau = (n+1) \cdot 0.17\,s$, with $n = 0, 13, 25, 50, 100$.

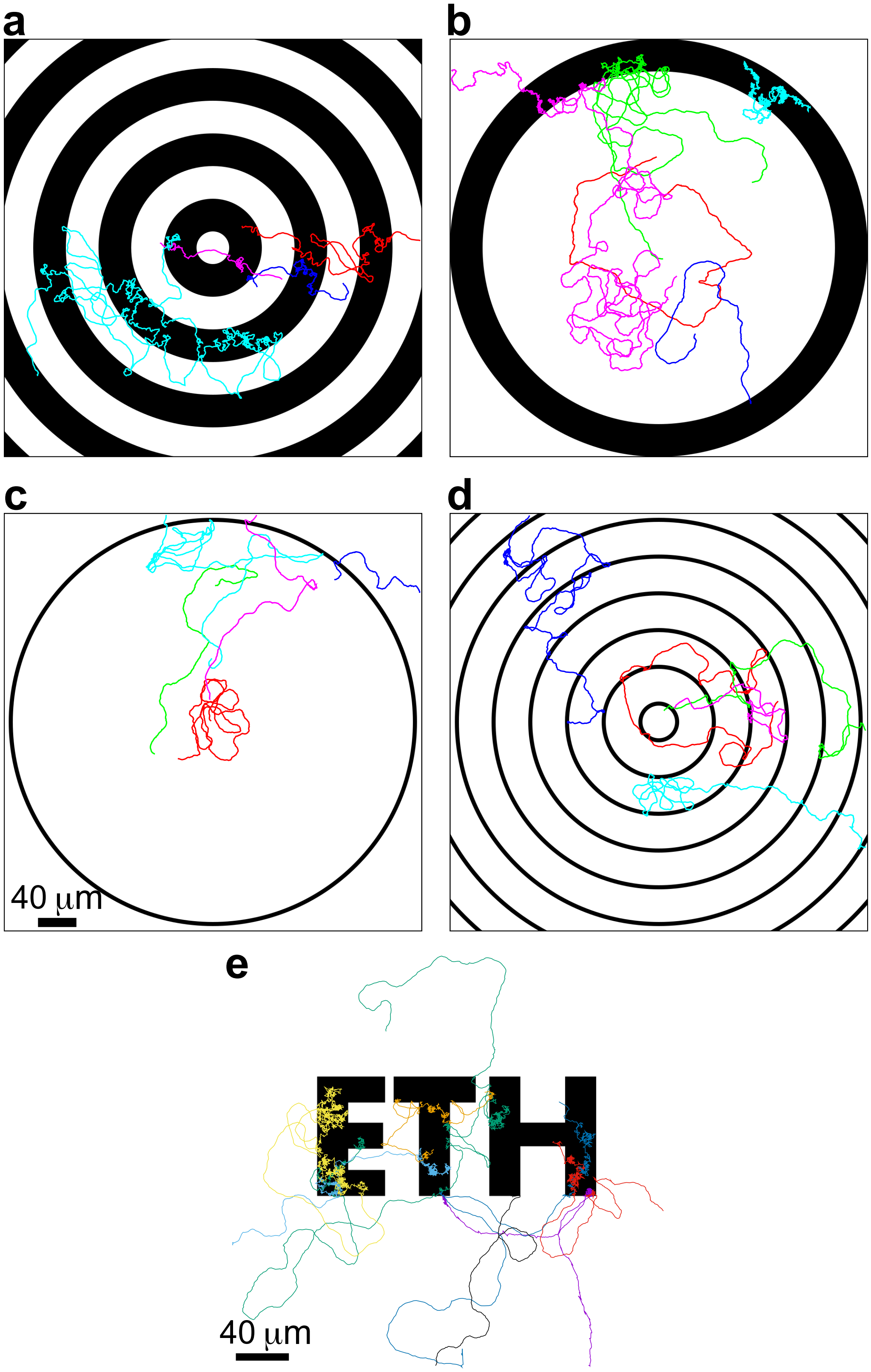

Supplementary Figure 6. Experimental trajectories of particles moving over arbitrary D_R landscapes. **a-d** v=1-5 μm s^{-1} and $\tau = 0.4\,s$. The white background corresponds to a D_R of 0.07 s^{-1} and the black background to a D_R of 1.2 s^{-1}. **e** $v \simeq 3\,\mu m/s$ and $\tau = 0.4\,s$. The white background corresponds to a D_R of 0.1 s^{-1} and the black background to a D_R of 10 s^{-1}.

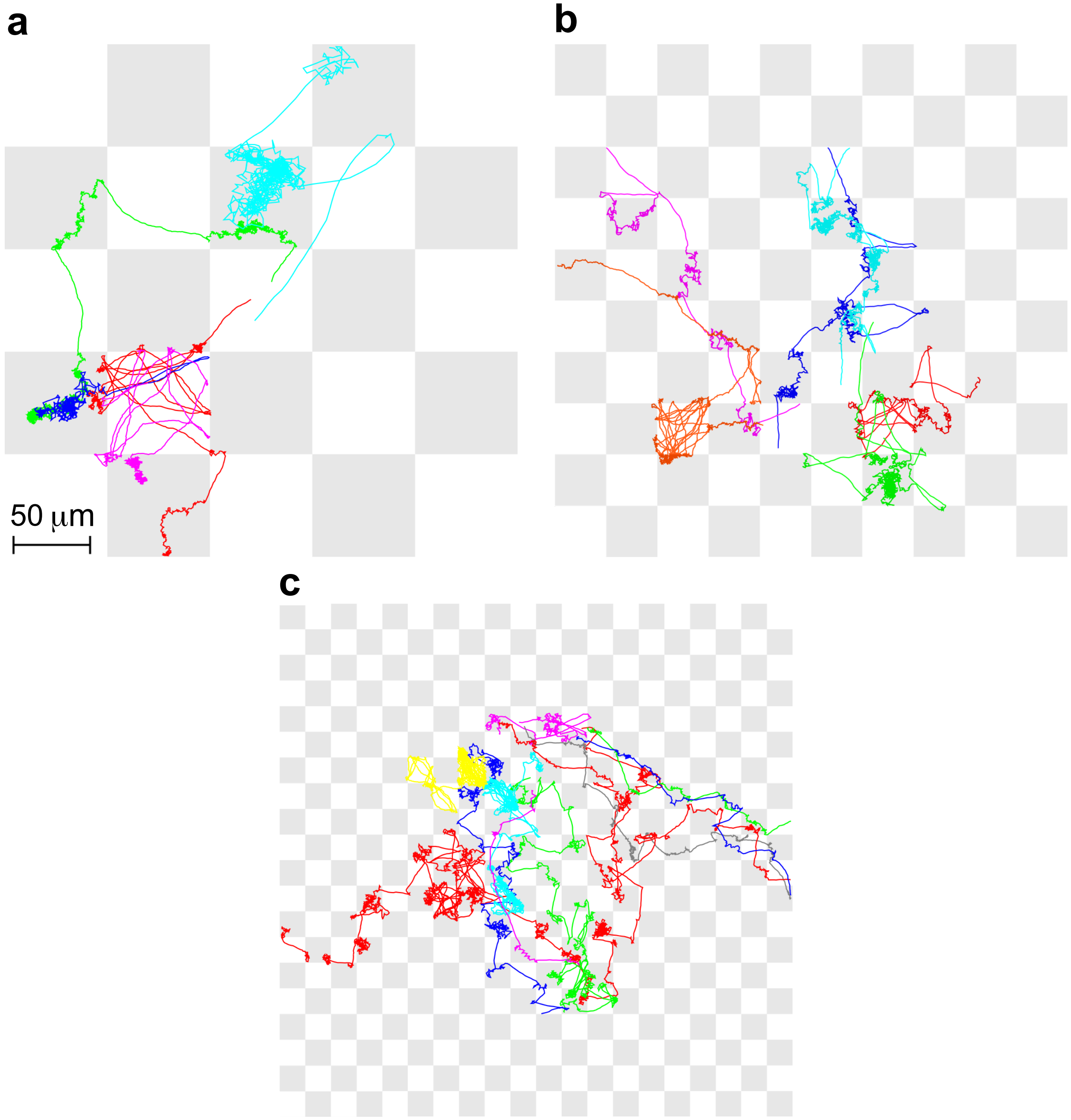

Supplementary Figure 7. Experimental trajectories of $4\,\mu m$ Janus particles with D_{R} varying according to checkerboard patterns with different tile sizes (L) and $\tau = 0.4\,s$: **a** $L = 64\,\mu m$, **b** $L = 32\,\mu m$, and **c** $L = 16\,\mu m$.

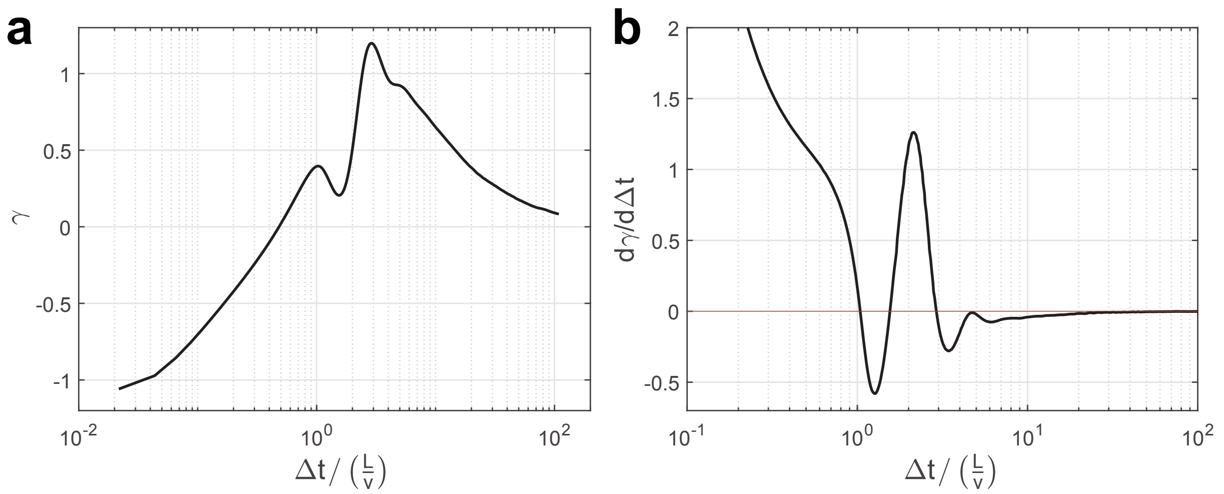

Supplementary Figure 8. Non-Gaussianity of the distribution of one-dimensional displacements $G(x, \Delta t)$ of an active Brownian particle with $D_{\rm R}$ varying according to the checkerboard pattern of Figure 2 for $\tau = 0$, $L = 32$ μm and $v = 3.5$ μm s^{-1}. **a**, Excess kurtosis γ of the simulated $G(x, \Delta t)$ and **b** its derivative with respect to Δt.
