# Supplementary material for: Feedback-controlled active brownian colloids with space-dependent rotational dynamics
Source: Nat Commun. 2020 Aug 24;11:4223. doi: 10.1038/s41467-020-17864-4 (PMC7445303; doi:10.1038/s41467-020-17864-4)
Supplement: Supplementary file 3 — Description of Additional Supplementary Files [file 41467_2020_17864_MOESM3_ESM.pdf]

# Description of Additional Supplementary Files

- **Supplementary Movie 1.** Manual control on the direction of propulsion of a 4 µm Janus particle (active vs. Brownian motion).
- **Supplementary Movie 2.** 4 µm Janus particle with different imposed $D_R$ = 0.1, 1, 5, 10 $s^{-1}$.
- **Supplementary Movie 3.** 4 µm Janus particle moving with a fixed AC voltage of 5 V (1 kHz) and a $D_R$ that increases over time in a step-wise fashion according to the progression: 0.1, 1, 5, 10 $s^{-1}$.
- **Supplementary Movie 4.** 4 µm Janus particle moving with a constant $D_R$ = 1 $s^{-1}$ but varying $v$ as the AC voltage is increased over time in a step-wise fashion between 3 and 9 V (1 kHz).
- **Supplementary Movie 5.** 4 µm Janus particles with $D_R$ varying according to a checkerboard pattern: $D_R$ is 0.01 $s^{-1}$ or 10 $s^{-1}$ when the Janus particle is moving over white or black tiles, respectively. The AC electric field is fixed at 6 V (1 kHz) and $\tau$ = 0.4 s.
- **Supplementary Movie 6.** Simulated trajectories of active Brownian particles with $D_R$ varying according to a checkerboard pattern as in Supplementary Movie 5 for different values of $\tau$. The simulation parameters are L = 32 µm and $v$ = 4 µm/s.
- **Supplementary Movie 7.** Simulated evolution of the positions of active Brownian particles with $D_R$ varying according to a checkerboard pattern as in Supplementary Movie 5 for different values of $\tau$. The simulation parameters are L = 32 µm and $v$ = 4 µm/s.
